# Impact of lockdown on children with type-1 diabetes: returning to the community was associated with a decrease in HbA1c

**DOI:** 10.3389/fped.2023.1245861

**Published:** 2023-12-20

**Authors:** Benjamin Morat, Nadine Lucidarme, Auriane Gibert, Carole Harbulot, Noémie Lachaume, Stéphanie Gréteau, Romain Basmaci

**Affiliations:** ^1^ Service de Pédiatrie-Urgences, AP-HP, Hôpital Louis-Mourier, Colombes, France; ^2^ Service de Pédiatrie Générale, AP-HP, Hôpital Jean-Verdier, Bondy, France; ^3^ Université Paris Cité, Inserm, IAME, Paris, France

**Keywords:** diabetes mellitus, lockdown, children, HbA1c, pediatrics, hypoglycemia, SARS-CoV-2, COVID-19

## Abstract

**Background:**

In March 2020, a 2-month lockdown of the entire population has been declared in France to limit the spread of COVID-19. Sudden changes in daily life can impact the glycemic control of patients with type 1 diabetes (T1D), especially children and adolescents. We aimed to assess the impact of the lockdown on glycemic control in children and adolescents with T1D.

**Methods:**

Children with T1D were prospectively recruited in two pediatric centers from May 11 to August 1, 2020. At inclusion, patients and/or parents were asked to fill in a form assessing the patient's lifestyle during the lockdown and a medical case report form was filled in by clinician. The mean of the three last glycated hemoglobin (HbA1c) values obtained before lockdown (HbA1c_mean; before March 17, 2020) was compared to the first HbA1c value measured after the lockdown (HbA1c_after; from May 11 to August 1, 2020). Univariable and multivariable analyses were performed, as appropriate, to identify factors associated with glycemic changes during lockdown.

**Results:**

One-hundred-and-eighteen children and adolescents (median age was 14.1 years, 50% males) with T1D (median time from diagnosis was 4.1 years) were enrolled in the study. No significant difference was observed between medians of HbA1c_mean and HbA1c_after values (8.37% [7.88; 9.32%] vs. 8.50% [7.70; 9.50%], respectively; *p* = 0.391). Returning to the community was a protective factor [OR 0.31 (0.09–0.94); *p* = 0.045]. Patients having increased HbA1c were more frequently in contact with a suspected case of COVID-19 [OR 9.07 (2.15–53.66); *p* = 0.006], whereas patients having decreased HbA1c had the feeling of increase number of hypoglycemia [OR 0.19 (0.05–0.57); *p* = 0.006].

**Conclusion:**

In our patients, HbA1c before and after the lockdown was stable. In subgroup analysis, returning to the community was a protective factor. In addition, feeling of hypoglycemia was more frequent in the patients with decreased HbA1c.

## 1. Introduction

On 30 January 2020, the World Health Organization officially declared the Severe Acute Respiratory Syndrome-CoronaVirus-2 (SARS-CoV-2) a risk to world public health and on March 11, 2020 declared coronavirus disease 19 (COVID-19) a pandemic health emergency (1).

In France, a lockdown has been declared from March 17 to May 10, 2020. In order to prevent the spread of COVID-19, the government announced strong restrictive measures on people's daily activities and movements, only essential services were guaranteed (2–4). Stay-at-home recommendations were reinforced for people with diabetes.

Prior to COVID-19, there have been few situations comparable to lockdown, but some data on glycemic control in diabetic patients during restrictions due to war or natural disasters show a significant increase in HbA1c (5).

The social distancing measures led to a sudden change of daily life, which may have had a great influence on diabetes management in children and adolescents with T1D. Indeed, the amount of time spent at home, with or without close parental supervision, may have increased compliance and glycemic control, however, due to the short-term notification, children of parents with essential jobs had to attend daycare institutions without diabetes-trained personnel. Moreover, glycemic control in children and adolescents with T1D may have deteriorated by effects of the lockdown on the patients' psychological wellbeing (6), by diminished utilization of healthcare measures, or by reduced parental guidance. Several international multicentric studies in non-diabetic adolescents have noted a reduction in physical activity (7) and changes in nutritional habits (8), which has been also observed in young people with diabetes.

In this study, we aimed to analyze the impact of the lockdown on glycemic control in children and adolescents with T1D and the factors associated with its possible change.

## 2. Patients and methods

### 2.1. Study design and participants

We performed a prospective observational cohort study in two academic pediatric centers nearby Paris, France. Patients were eligible if (i) they were aged of 18 years old or less at enrolment; and (ii) they had a clinical diagnosis of T1D diagnosed before June 2019. Patients were prospectively recruited during a hospitalization or an outpatient clinic appointment from May 11 to August 1, 2020. Exclusion criteria were: a hospitalization for changing the treatment regimen in the 6 months prior to March 17, 2020; the absence of 3 values of HbA1c before the lockdown; the absence of a value of HbA1c during the recruitment period; and patients or parents who declared their opposition to collect the data.

### 2.2. Procedures

At enrolment, patients and their parents or legal guardians were asked to complete a form assessing their lifestyle during the lockdown (number and lifestyle of household members, respect of the rules of social distancing, sport, food and sleeping habits), whether they were in contact with a suspected or confirmed case of COVID-19, and their feeling about their glycemic control (Supplementary Material S1).

A second case report form has been created and completed for each patient by the pediatrician in charge, including clinical data, medical background, history of T1D, biologic and therapeutic data before and after lockdown (Supplementary Material S2).

### 2.3. Outcomes

The primary outcome was the evolution of the glycated hemoglobin (HbA1c) before and after lockdown by comparison between the mean of the three HbA1c before lockdown (HbA1c_mean) and the value of the first HbA1c after lockdown (HbA1c_after). The main secondary outcomes were the differences between features observed before and during/after lockdown, such as: the number of severe hypoglycemia (defined as hypoglycemia requiring assistance due to altered consciousness) and of hyperglycemia with ketonemia or diabetic ketoacidosis; the total daily dose of insulin; the proportion of time spent in the target range (TIR; i.e., 70–180 mg/dl), below the target range (TBR; i.e., less than 70 mg/dl), and above the target range (TAR; i.e., more than 180 mg/dl) for patients withflash glucose monitoring, which is a type of continuous glucose monitoring that needs to be intermittently scanned to access at the glucose levels. This system continuously samples and measures interstitial glucose levels; a new glucose value is generated each minute. The sensor can provide glucose values for 14 days if the patient scans at least every 8 h. If not, the glucose information from the previous 8-hour period is deleted. We also described the changes in lifestyle during lockdown.

### 2.4. Subgroups

We decided to separate the whole population in two different groups regarding on evolution between HbA1c_mean and HbA1c_after (ΔHbA1c = HbA1c_after—HbA1c_mean). The first group would consist of all patients with ΔHbA1c < 0 (improvement of glycemic control) while the second would consist of all patients with ΔHbA1c > 0 (degradation of glycemic control). For the statistical analysis, it was decided to exclude patients with a strictly stable HbA1c (ΔHbA1c = 0). These subgroups were compared to identify some factors associated with HbA1c variability.

### 2.5. Statistical analysis

Results are shown as medians (interquartiles) for continuous variables and numbers (percentages) for categorical variables. Wilcoxon test and Fischer exact test were used as appropriate to assess differences between independent subgroups; a paired Wilcoxon test was used as appropriate to assess the differences between subjects before and after the lockdown.

All variables identified on univariate analysis as potential factors associated with increased HbA1c levels (*p* < 0.20) were introduced in a binary logistic regression model to estimate odds ratios (ORs) and 95% confidence interval (CI). A step-by-step approach was further performed to identify the best model. A *p*-value < 0.05 was considered statistically significant. All the tests were performed using R statistical and forestmodel packages, version 4.0.3 [R Project for Statistical Computing (RRID:SCR_001905)].

### 2.6. Ethics

According the French national policy, an information letter was given to the parents or legal guardians and the non-opposition was recorded. Patients were excluded if the opposition of collecting or using data was expressed.

The study protocol has been approved by the Robert-Debré Ethics Committee (IRB 00006477) under number 2020-517.

## 3. Results

### 3.1. Patients' characteristics

**Figure 1:**
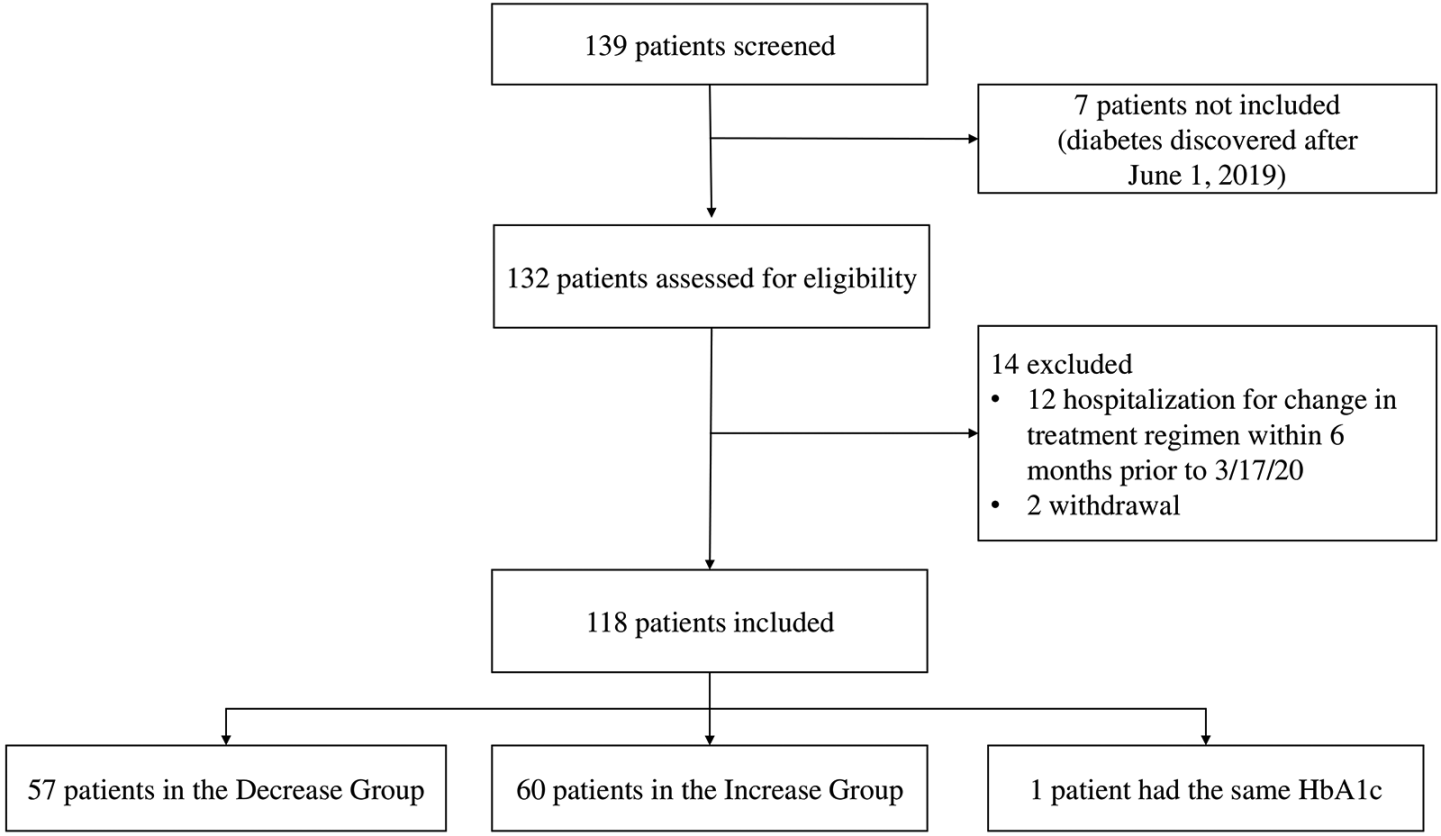
Flowchart of the diabetic pediatric patients.

During the study period, 139 individuals were screened, and 118 were enrolled (Figure 1). Demographic and clinical characteristics of the population are shown in Table 1. Participants were aged from 4 to 18 years, with a median age of 14.1 years at recruitment, 67% were older than 12 years old. Median duration of diabetes since diagnosis was 4.1 years. Most of our patients were on a multiple injection therapy (*n* = 109; 92%) and had a Continuous Glucose Monitoring (CGM) (*n* = 95; 81%). Ninety-five patients (81%) had no comorbidities other than diabetes and 111 patients (94%) had no treatment other than insulin (Table 1).

**Table 1:** Demographic and clinical features of the patients.

Characteristics of patients	Total (*n* = 118)	Decrease group (*n* = 57)	Increase group (*n* = 60)	*p*-value
Age—Years	14.1 (10,9; 15.9)	14.1 (10.2; 15.6)	14.2 (11.1; 16.1)	0.522
Sex—No (%)				
Female	59 (50)	28 (49)	30 (50)	1
Male	59 (50)	29 (51)	30 (50)	
Weight—kg				
Before lockdown	51.80 (36.8; 63.0)	49.5 (35.1; 62.3)	54.7 (42.7; 65.0)	0.200
After lockdown	54.6^a^ (38.7; 65.0)	52.0^a^ (36.3; 64.0)	56.8^a^ (43.0; 65.7)	0.219
Weight evolution	+1.9 (0.6; 4.2)	+1.7 (0.6; 3.8)	+2.0 (0.7; 4.6)	0.525
Weight—Standard deviation				
Before lockdown	1.45 (0.40; 2.34)	1.15 (0.34; 1.92)	1.74 (0.45; 2.50)	0.099
After lockdown	1.49^b^ (0.42; 2.44)	1.27^c^ (0.19; 1.97)	1.82^d^ (0.60; 2.66)	0.143
Weight evolution	+0.08 (−0.11;+0.36)	+ 0.02 (−0.09;+0.33)	+ 0.10 (−0.13;+0.40)	0.720
T1D duration—Years	4.1 (2.6; 8.4)	4.2 (2.8; 9.2)	4.1 (2.5; 7.9)	0.547
Insulin therapy—No (%)				
Multiple injection	109 (92)	52 (91)	57 (95)	0.483
Pump	9 (8)	5 (8)	3 (5)	
Continuous Glucose Monitoring—No (%)	95 (81)	46 (81)	48 (80)	1
Carbohydrate counting—No (%)	6 (5)	3 (5)	2 (3)	0.674
Comorbidities—No (%)				
None	95 (81)	47 (82)	47 (78)	0.646
One or more	23 (19)	10 (18)	13 (22)	

Results are shown as medians (interquartiles) except when specified.

T1D, type 1 diabetes.

^a^ *p* < 0.001.

^b^ *p* = 0.003.

^c^ *p* = 0.069.

^d^ *p* = 0.031.

### 3.2. Primary outcome

**Figure 2:**
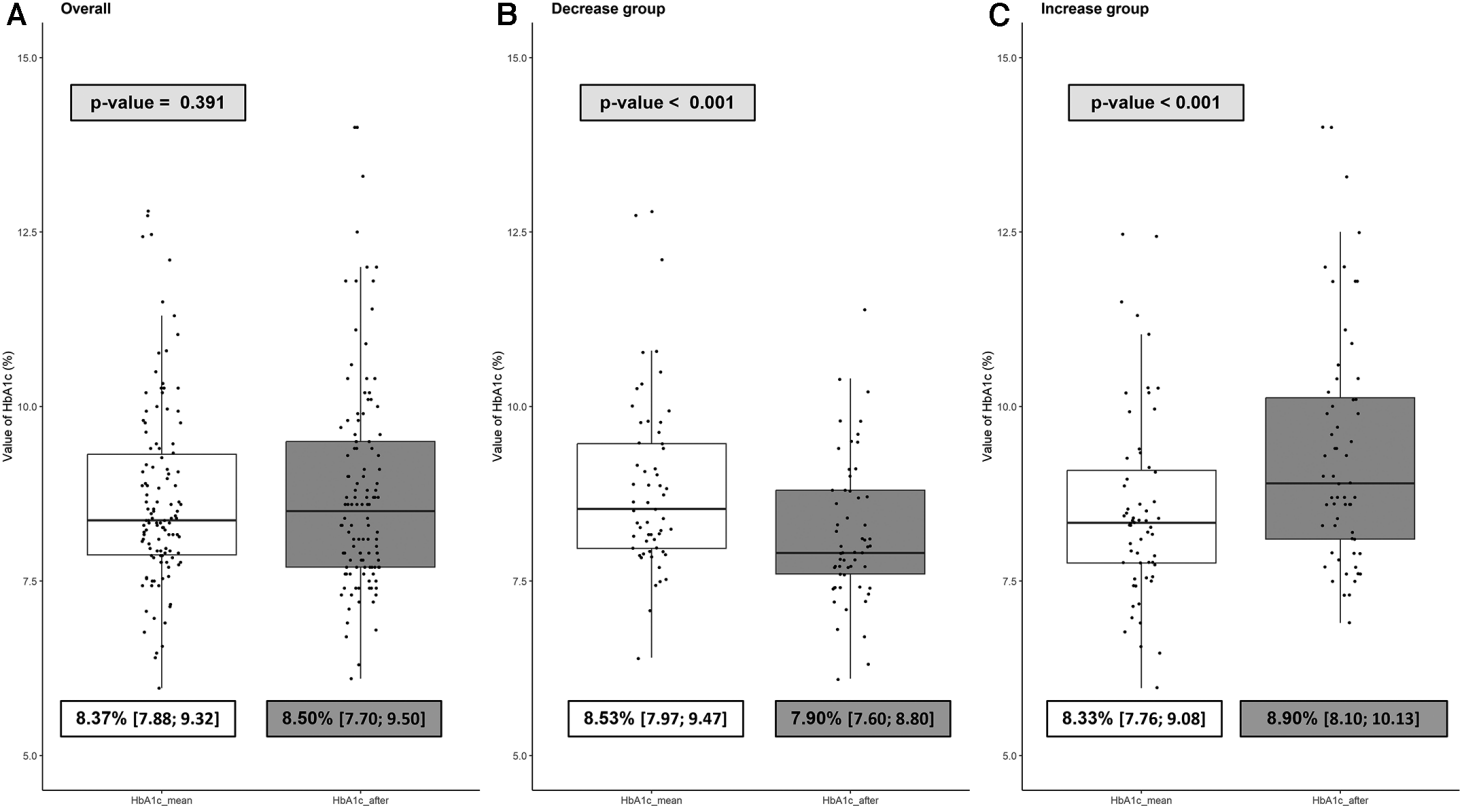
Boxplot of the evolution of HbA1c before and after the lockdown in the overall population (**A**), the decrease group (**B**) and the increase group (**C**). HbA1c_mean represents the mean of the three HbA1c before lockdown. HbA1c_after represents the value of the first HbA1c after lockdown. ΔHbA1c = HbA1c_after—HbA1c_mean. The decrease group consists of all patients with ΔHbA1c < 0 (improvement of glycemic control) while the increase group consists of all patients with ΔHbA1c > 0 (degradation of glycemic control).

The primary outcome was the evolution of HbA1c before and after lockdown (Figure 2A). No significant difference was observed between HbA1c_mean and HbA1c_after values in the entire population (median 8.37% [IQR = 7.88; 9.32] vs. 8.50% [7.70; 9.50], respectively; p = 0.391).

Further, we identified two subgroups: the “decrease group” (*n* = 57; 48%), with ΔHbA1c < 0 and the “increase group” (*n* = 60; 51%), with ΔHbA1c > 0. Evolution of HbA1c for these groups is shown in Figures 2B,C. As expected, HbA1c_after was significantly lower than HbA1c_mean in the decrease group (7.90% [7.60; 8.80] vs. 8.53% [7.97; 9.47], respectively; *p* < 0.001 with a median variation of −0.67% [−0.97; −0.27] (Figure 2B). On the other hand, HbA1c_after was significantly higher than HbA1c_mean in the increase group (8.90% [8.10; 10.13] vs. 8.33% [7.76; 9.08], respectively; *p* < 0.001) with a median variation of +0.70% [0.33; 1.28] (Figure 2C).

### 3.3. Comparison of data before and after the lockdown

We described the evolution of the weight, the parameters of diabetes survey and treatment before and after the lockdown in the entire population.

Overall, patients significantly gained weight during this period [+1.90 kg (0.60; 4.20); *p* < 0.001], with a median time of 4.6 (3.7–5.3) months between both measures, that was confirmed in standard deviation to avoid the effect of the normal growth of children [+0.08 SD (−0.11; +0.36), *p* = 0.003] (Table 1).

Table 2 shows the evolution of the daily dose of insulin and the parameters assessing diabetes equilibrium between the periods before and after lockdown. After the lockdown, we observed a significant increased number of flash performed per day over the last 90 days (*p* < 0.001), and a higher proportion of glycemic data captured over the last 90 days (*p* = 0.002). Those characteristics were similarly observed in the increase and decrease groups (Table 2).

**Table 2:** Evolution of diabetes parameters before and after lockdown.

Diabetes parameters	Total (*n* = 118)			Decrease group (*n* = 57)			Increase group (*n* = 60)		
	Before lockdown	After lockdown	*p*-value	Before lockdown	After lockdown	*p*-value	Before lockdown	After lockdown	*p*-value
Total insulin—IU/kg/d	0.94 (0.70; 1.09)	0.90 (0.75; 1.10)	0.425	0.96 (0.80; 1.10)	0.88 (0.76; 1.11)	0.306	0.87 (0.645; 1.070)	0.9 (0.74; 1.08)	0.080
Severe hypoglycemia within 3 months—No	0 (0; 0)	0 (0; 0)	**/**	0 (0; 0)	0 (0; 0)	**/**	0 (0; 0)	0 (0; 0)	**/**
Hyperglycemia with ketosis within 3 months—No	0 (0; 0)	0 (0; 0)	–	0 (0; 0)	0 (0; 0)	–	0 (0; 0)	0 (0; 0)	–
Mean Glycemia 30 days—mg/dl	198 (177; 223)	199 (171; 225)	0.491	193 (181; 223)	194 (166; 213)	0.688	201 (172; 223)	207 (181; 232)	0.711
Mean Glycemia 90 days—mg/dl	200 (185; 227)	201 (174; 231)	0.920	198 (181; 229)	187 (171; 211)	**0**.**050**	203 (185; 227)	209 (181; 256)	**0**.**035**
Flash per day 30 days—No	4 (2; 7)	5 (3; 9)	0.490	4 (3; 7)	7 (3;10)	0.866	4 (1; 6)	5 (2; 7)	0.396
Flash per day 90 days—No	3 (1; 5)	6 (4; 10)	**<0**.**001**	4 (2; 5)	7 (4;11)	**0**.**004**	2 (1; 5)	6 (3; 9)	**0**.**024**
Data recorded 30 days—%	79 (45; 94)	77 (50; 95)	0.639	72 (46; 93)	82 (50; 96)	0.809	81 (50; 96)	74 (50; 90)	0.353
Data recorded 90 days—%	52 (32; 64)	79 (52; 95)	**0**.**002**	42 (26; 60)	79 (47; 96)	0.064	58 (34; 64)	78 (56; 90)	**0**.**018**

Results are shown as median (interquartiles) except when specified. IU, international units; statistically significant differences are shown in bold font.

**Figure 3:**
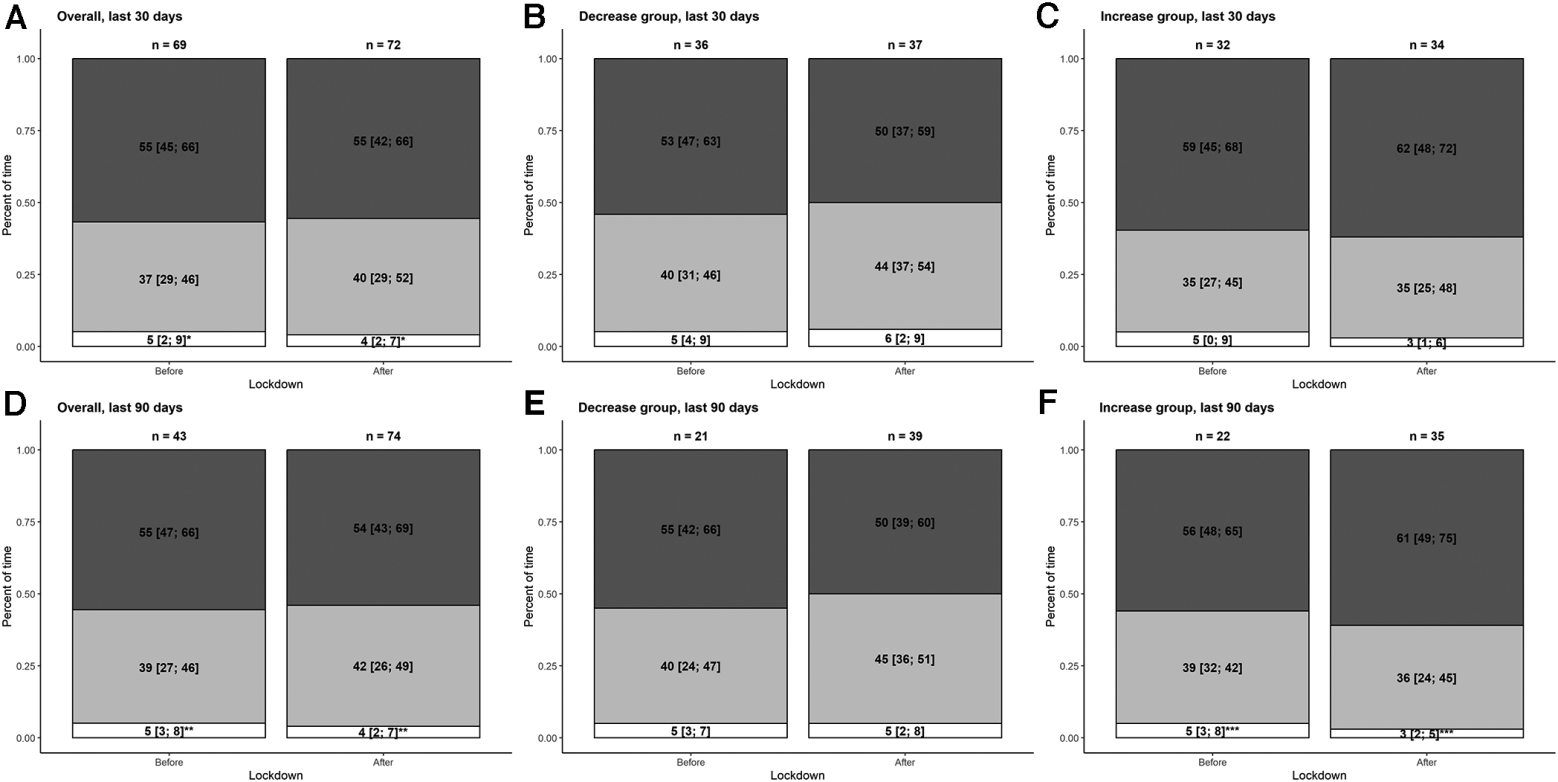
Barplot of the flash glucose monitoring data in the overall population (**A** and **D**), the decrease group (**B** and **E**) and the increase group (**C** and **F**). Proportions (in %) of time spent below the target range (less than 70 mg/dl), within the target range (70–180 mg/dl) and above the target range (more than 180 mg/dl) are represented in white, light grey and dark grey, respectively. Results are shown as medians (interquartiles). The first row represents the data over the last 30 days. The second row represents the data over the last 90 days. The overall population, the decrease group, and the increase group are represented on the left-hand, middle and right-hand sides, respectively. *p = 0.026; **p = 0.038; ***p = 0.016.

Figure 3 represents the proportion of time spent below, in and above the glycemic range (70 to 180 mg/dl). A significant lower proportion of TBR over the last 30 days (*p* = 0.026) or 90 days (*p* = 0.038) in the entire population was observed, but those characteristics were (or tended to be) only significant in the increase group, as expected. No other variables were statistically significant between both periods in the entire population. As expected, the mean blood glucose level over the last 90 days tended to be lower after lockdown in the decrease group (187 mg/dl [171–211] vs. 198 mg/dl [181–229], *p* = 0.050), while it was significantly higher in the increase group (209 mg/dl [181–256] vs. 203 mg/dl [185–227], *p* = 0.035) (Table 2).

### 3.4. Subgroups analysis

We attempted to identify factors associated with HbA1c levels variability after lockdown.

They were no significant differences between the increase group and the decrease group regarding age, gender, center, weight, duration of diabetes, treatment regimen or its change within a year, carbohydrate counting, type of glucose monitoring, comorbidities and treatment other than insulin (Table 1).

However, we observed some significant differences in the lifestyle between both groups (Tables 3–5). More patients returned to the community in the decrease group than in the increase group (*n* = 15 [28%] vs. *n* = 6 [11%], respectively; *p* = 0.029) during the study period. Although few, we observed that more patients or family living at home in the increase group were in contact with a suspected case of COVID-19 (21% vs. 6%; *p* = 0.026) (Table 3). Finally, patients in the decrease group were more likely to report a feeling of an increase number of hypoglycemia (38% vs. 14%; *p* = 0.008) (Table 5). The rest of the characteristics concerning the lifestyle during lockdown are detailed in Tables 3–5 and found no other significant differences between both groups.

**Table 3:** Behavior and COVID history during the lockdown.

Characteristics	*n*	Total (*n* = 118)	*n*	Decrease group (*n* = 57)	*n*	Increase group (*n* = 60)	*p*-value
Children at home—No	118	2 (1; 3)	57	2 (1; 3)	60	2 (1; 3)	0.412
Adults at home -No	109	2 (2; 3)	53	2 (2; 3)	55	2 (2; 3)	0.716
Father's professional activity—No (%)							
No activity	81	37 (46)	41	17 (41)	39	20 (51)	0.850
Work at office		22 (27)		12 (29)		10 (26)	
Telework		20 (25)		11 (27)		8 (21)	
Mixed		2 (2)		1 (2)		1 (3)	
Mother's professional activity—No (%)							
No activity	98	62 (63)	49	33 (67)	48	28 (58)	0.763
Work at office		16 (16)		8 (16)		8 (17)	
Telework		18 (18)		7 (14)		11 (23)	
Mixed		2 (2)		1 (2)		1 (2)	
Siblings in a community—No (%)	110	4 (4)	53	1 (2)	56	3 (5)	0.619
Patient returned to community—No (%)	111	21 (19)	54	15 (28)	56	6 (11)	**0**.**029**
COVID at home—No (%)							
Evocative symptoms	117	18 (15)	56	7 (13)	60	11 (18)	0.448
Positive PCR test	17	4 (24)	7	2 (29)	10	2 (20)	1
Contact with a suspected case	112	15 (13)	53	3 (6)	58	12 (21)	**0**.**026**
Contact with a PCR positive case	114	12 (11)	55	3 (5)	58	9 (16)	0.126

Results are shown as median (interquartiles) except when specified. Statistically significant differences are shown in bold font.

**Table 4:** Sports, sleep and food habits during the lockdown.

Characteristics	*n*	Total (*n* = 118)	*n*	Decrease group (*n* = 57)	*n*	Increase group (*n* = 60)	*p*-value
Sports compared to before lockdown—No (%)							
Less often	109	73 (67)	53	35 (66)	55	38 (69)	0.387
In the same way		15 (14)		6 (11)		9 (16)	
More often		11 (10)		8 (15)		3 (5)	
Do not know		10 (9)		4 (8)		5 (9)	
Sports sessions per week—No (%)							
0 to 1	116	66 (57)	55	30 (55)	60	35 (58)	0.878
2 to 3		32 (28)		15 (27)		17 (28)	
4 to 6		8 (7)		5 (9)		3 (5)	
7 or more		10 (9)		5 (9)		5 (8)	
Change in sleep pattern—No (%)	117	105 (90)	56	49 (88)	60	55 (92)	0.549
Balanced diet—No (%)	109	84 (77)	53	44 (83)	55	40 (73)	0.249
Increased carbohydrate intake—No (%)	109	51 (47)	52	25 (48)	56	25 (45)	0.847
Snacking per week—No (%)							
0	114	34 (30)	55	16 (29)	58	18 (31)	0.576
1 to 3		46 (40)		25 (45)		21 (36)	
4 to 7		18 (16)		9 (16)		9 (16)	
8 or more		16 (14)		5 (9)		10 (17)	

Results are shown as median (interquartiles) except when specified. Statistically significant differences are shown in bold font.

**Table 5:** Diabetes survey during the lockdown.

Characteristics	*n*	Total (*n* = 118)	*n*	Decrease group (*n* = 57)	*n*	Increase group (*n* = 60)	*p*-value
Feeling of poorer glycemic control—No (%)	113	72 (64)	53	30 (57)	59	41 (69)	0.174
Feeling of increased hypoglycemia—No (%)	111	28 (25)	53	20 (38)	57	8 (14)	**0**.**008**
Feeling of increased hyperglycemia—No (%)	114	79 (69)	54	36 (67)	59	42 (71)	0.685
Contact of the patient by the physician—No (%)	118	74 (63)	57	40 (70)	60	34 (57)	0.179
Contact of the physician by the patient—No (%)	117	42 (36)	56	21 (38)	60	21 (35)	0.848
Hospitalization—No (%)							
In total	118	16 (14)	57	8 (14)	60	8 (13)	1
Change in treatment regimen		5 (4)		1 (2)		4 (7)	0.223
Imbalance		6 (5)		3 (5)		3 (5)	
Annual check-up		5 (4)		4 (7)		1 (2)	

Results are shown as median (interquartiles) except when specified. Statistically significant differences are shown in bold font.

**Figure 4:**
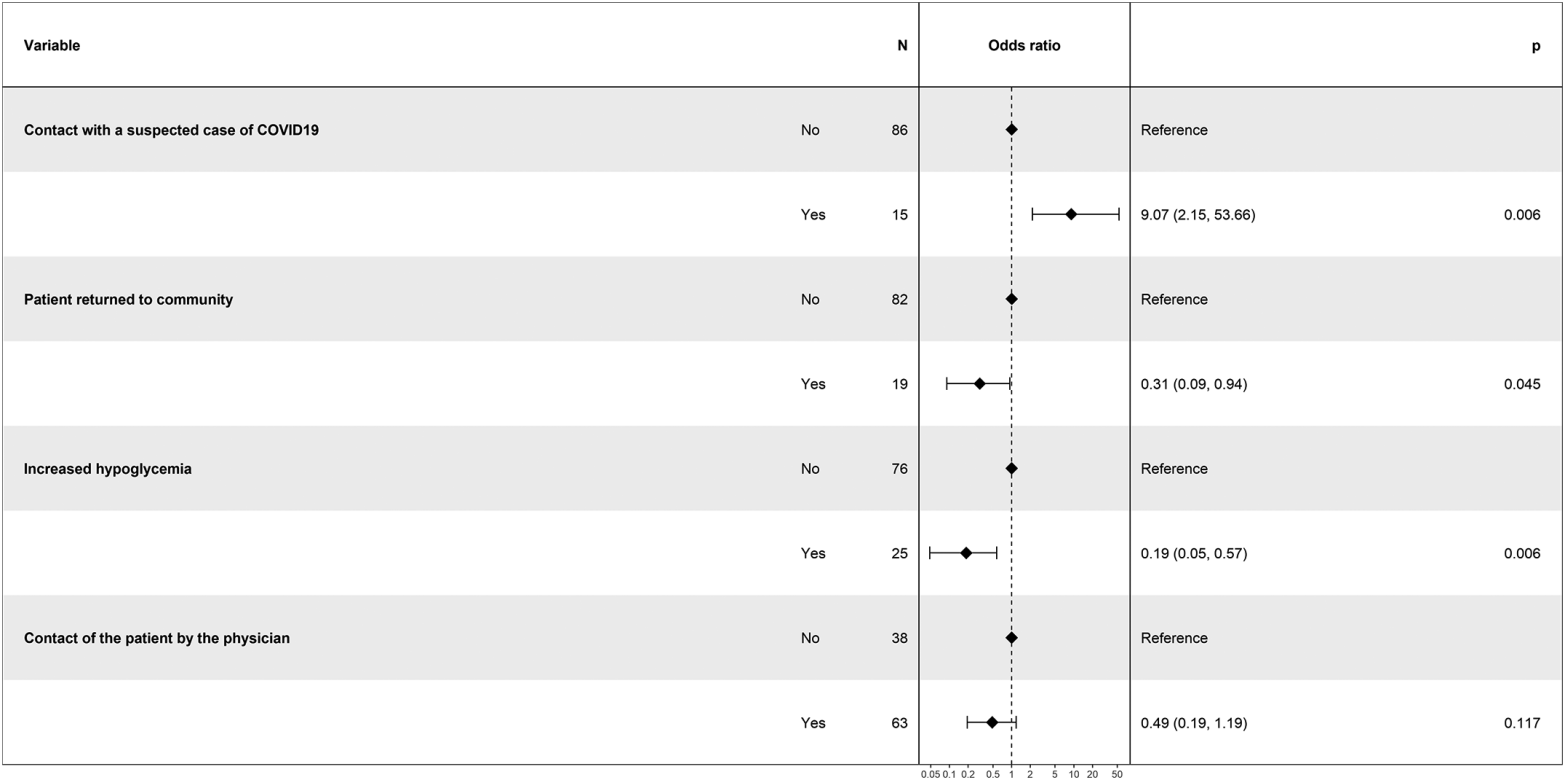
Forestplot of the multivariable analysis to assess factors associated with increased HbA1c levels during lockdown. Results are shown as odds ratio (95% confidence interval).

In order to identify independent factors associated with increased HbA1c levels during lockdown, we performed a multivariable analysis. The step-by-step selection model allowed to identify that the contact with a suspected case of COVID-19 was associated with an increase in HbA1c levels [OR 9.07 (2.15–53.66); *p* = 0.006], whereas returning to the community [OR 0.31 (0.09–0.94); *p* = 0.045] and the feeling of increase number of hypoglycemia [OR 0.19 (0.05–0.57); *p* = 0.006] were associated with a decrease in HbA1c. Finally, patients contacted by their physician tended to have a better glycemic control [OR 0.49 (0.19–1.19); *p* = 0.117] (Figure 4).

## 4. Discussion

In this large series assessing glycemic control in children with T1D, we did not observe a significant difference between HbA1c_mean before lockdown and the HbA1c_after. On the one hand, we could have expected an increase in HbA1c with the confinement, in particular due to the decrease in physical activities, the degradation of the food balance and the change of the nychthemeral rhythm. On the other hand, some factors could have led to a better glycemic control since we observed during/after the lockdown a significant increased number of flash performed per day over the last 90 days (*p* < 0.001), and a higher proportion of glycemic data captured over the last 90 days in both groups. That could be explained by the possibility to take care of the diabetes more frequently when other daily activities were no longer available (sports, school, outings…). However, despite this stronger monitoring of glucose levels, it seems that it was not sufficient to influence positively the glycemic control assessed with HbA1c.

Two distinct populations appeared in our study, one population with worsening glycemic balance (+0.7%) and another with improving glycemic balance (−0.67%). The multivariable analysis identified two factors associated with a decrease in HbA1c during lockdown: returning to the community [OR 0.31 (0.09–0.94); *p* = 0.045] and the increase number of hypoglycemia feelings [OR 0.19 (0.05–0.57); *p* = 0.006]. We can hypothesize that returning to the community may improve glycemic control by improvement of eating habits or increased daily exercise. French pediatricians promoted early the school reopening during COVID-19 pandemic, and schools remained open more frequently than in numerous other countries (3, 4). As described since 1993 with the publication of the Diabetes Control and Complications Trial Research Group (9), the improvement in glycemic control is related to the use of diabetic intensive therapy and its use outweigh the increased risk of hypoglycemia that accompanies such treatment. The frequent hypoglycemia feeling can be considered as a marker of the reduction achieved in HbA1c. The data associated with blood glucose sensor (TBR, TIR, TAR) in our study included too much missing data to be statistically significant but other pediatric studies have found no difference in TBR before and after lockdown (10, 11).

The risk factor for increased HbA1c levels was a contact with a suspected case of COVID-19 for the patient or a member of the family living at home [OR 9.07 (2.15–53.66); *p* = 0.006]. This contact may have led to undiagnosed SARS-CoV-2 infections (few tests were available and performed at that time, especially without symptoms). An Italian study demonstrated the presence of new-onset hyperglycemia, insulin resistance and beta cell hyperstimulation in patients with COVID-19 without a history of diabetes (12). Stress which develops during infection or due to other factors (e.g., fear or worries of COVID-19) (13) can dysregulate glycemic control in non-diabetic individuals [stress hyperglycemia (14)], but can also be observed in patients with diabetes, with notably an increase in hyperglycemia (15). Coronavirus infections are proven to have a huge effect on the management of diabetes because they aggravate inflammation and alter immune system responses, leading to difficulties in glycemic control (16–18).

Of note, patients contacted by their pediatrician during lockdown tended to have a better glycemic control. With T1D, the development of numerous technologies with the transfer of data from continuous sensors and pumps to internet media is changing the way we approach the monitoring of patients with T1D. Some studies on the role of telemedicine during lockdown on glycemic control have shown a significant improvement in glycemic metrics (CGM data); supporting the clinical effectiveness of telemedicine in diabetes care (19–21).

In comparison, most other studies found no difference or a slight improvement in glycemic control before and after lockdown. In a French T1D adult population (1,378 individuals) with flash glucose monitoring devices, Potier et al. (22) found an improvement of the glycemic control during the lockdown, on average, from 163.5 to 155.7 mg/dl (p < 0.001). Similarly to our study, they identified as a factor associated with decreased HbA1c levels an increase in the frequency of flash glucose monitoring scans [OR 1.48 (1.04–2.10)], and the patients with better glycemic control had more frequent hypoglycemic events [OR 1.67 (1.13–2.46)], and an easier diabetes control perception [OR 1.71 (1.18–2.49)]. In the largest pediatric cohort published to date on 19,729 pediatric T1D patients, Hammersen et al. (23) found no clinically relevant difference in glycemic control before, during, and after the first lockdown in spring 2020 compared to the preceding year 2019. In another pediatric cohort (233 children and adolescents with T1D), Marigliano et al. (10) found after lockdown lower HbA1c (7.82 ± 0.84 vs. 7.44 ± 0.83, *p* < 0.001) and mean glucose (mg/dl) (178.6 ± 31.2 vs. 169.1 ± 28.6, *p* < 0.001). In a similar study, Tinti et al. (24) enrolled 66 children and adolescents, and found a mild but significant improvement of TIR (+2.8%), without any significant difference of TBR despite a significant reduction of the physical activity per week (6.1 ± 3.3 h to 2.7 ± 3.1 h). The patients in this study may have had an easier time adapting to changes thanks to two factors compared to our patients despite the less physical activity: 88% were carbohydrate counters and 55% were using an insulin pump (in our population, they were 5% and 8% only, respectively).

Lifestyle changes during lockdown were numerous. The increase in weight in all patients was significant (+1.90 kg [0.60; 4.20]; *p* < 0.001 and +0.08 SD [−0.11; 0.36]; *p* = 0.003) and was greater in the increase group than in the decrease group although the observed difference was not significant (+0.10 SD [−0.13; +0.40] vs. +0.02 SD [−0.09; +0.33], respectively; *p* = 0.720). The increase in weight in patients with diabetes can have important repercussions on therapeutics (insulin resistance) (25), however the weight in T1D can also be a marker of a good glycemic control through the insulin capacity to increase the lipogenesis in hepatocytes (26).

The decrease in sports, the change in sleep patterns and the increase in carbohydrate intake as well as significant snacking can probably explain this increase in weight in our population.

We did not evaluate physical activity but sport, which is only one part of the physical activity performed. A French study found a decrease in physical activity during confinement among children, in line with our study (27). A German study found that sports activity declined whereas recreational screen time increased (28). However, a substantial increase in habitual physical activities leads to an overall increase in physical activity among children and adolescents (28). A study with 280 Italian children with T1D also found a decline in sport (−2.1 ± 2.1 h/week) and outdoor-plays (−73.9 ± 93.6 min/day) during the lockdown (29).

The sleep habits of children were studied in an international study; lockdown was associated with later bedtime and wake time, this shift did not alter sleep duration in more than 40% of children (30).

Regarding food habits, an international study found adolescents also exhibited a higher sweet food consumption (8). Regarding children's eating behavior a Greek study found that the consumption of fruits and fresh fruit juices, vegetables, dairy products, pasta, sweets, total snacks, and breakfast significantly increased (31).

The main strengths of our study are that we included a diabetic pediatric population from two centers, and collected data on lifestyle during confinement and the patients' feelings about their diabetes.

The main weaknesses are the missing data regarding the collection of sensor data, some of the data are retrospective, and most of our patients were not at the recommended HbA1c target even before confinement. Moreover, subgrouping of samples into a group of HbA1c change below or more than zero is probably not an ideal model as slight changes above or below zero does not really differentiate between patients with poor or good outcome. Coexistence of children and adolescents may constitute a limitation due to differences in health behavior, although age did not appear as a factor influencing the glycemic control in our study. Our population was from the North of Paris with high frequency of disadvantaged social conditions, and then the results of this study may be not generalizable to other population. Finally, although our study included a significant number of patients for other pediatric studies, this number remains limited.

To conclude, we observed a quite stability or a slight improvement in glycemic control during first lockdown. In addition to factors associated with good outcome, our results may suggest that keeping children at school and doing teleconsultation may have a positive impact on glycemic control whether similar situation of lockdown should be established in future.

## Data Availability

The raw data supporting the conclusions of this article will be made available by the authors, without undue reservation.
