# Supplementary material for: Impact of lockdown on children with type-1 diabetes: returning to the community was associated with a decrease in HbA1c
Source: Front Pediatr. 2023 Dec 20;11:1245861. doi: 10.3389/fped.2023.1245861 (PMC10769491; doi:10.3389/fped.2023.1245861)
Supplement: Supplementary file 1 [file Datasheet1.pdf]

Lifestyle during the lockdown in children and adolescents with type 1

Identification : **Initials:** Last name: ___ First name: ___ Birth date __ __ / __ __ / __ __ __ __
Gender ☐M ☐F Date of questionnaire completion __ __ / __ __ / __ __ __ __

Lifestyle during the lockdown :

- How many people **regularly** lived in your home?
  - Number of children (under 18 years old) __ __ Number of adults (over 18 years old) __ __
- What professionnal activity did the adults in the home engage in ?
  - Father: ☐ Teleworking ☐ On-site work ☐ No work ☐ Not applicable
  - Mother: ☐ Teleworking ☐ On-site work ☐ No work ☐ Not applicable
  - Other adults: ☐ Teleworking (n=) ☐ On-site work (n=) ☐ No work (n=) ☐ Not applicable
- Did at least one of the children in the home go to community (nursery, school, etc.)? ☐ Yes ☐ No
  - If **yes**, how many days a week on average (for the child who goes most often): ☐ 1 ☐ 2-3 ☐ 4-5

Since May 11th 2020, has the young with diabetes been returned to the community (nursery, school, etc.)? ☐ Yes ☐ No

- If **yes**, specify the date of resumption of school or return to the community: __ __ / __ __ / __ __ __ __

Outings and COVID during the lockdown :

- On average, how many outings did you make outside the home **per week**?
  - Patient (Young with diabetes): ☐ 0-3 ☐ 4-7 ☐ 8-14 ☐ 15 or more
  - Father: ☐ 0-3 ☐ 4-7 ☐ 8-14 ☐ 15 or more ☐ Not applicable
  - Mother: ☐ 0-3 ☐ 4-7 ☐ 8-14 ☐ 15 or more ☐ Not applicable
  - Other person: ☐ 0-3 ☐ 4-7 ☐ 8-14 ☐ 15 or more ☐ Not applicable
- At least one person **living in the home** has experienced any of the following symptoms : fever, cough, loss of tast or smell, diarrhea, vomiting, difficulty breathing, chest pain, fatigue: ☐ Yes ☐ No
  - If **yes**, has this person tested **positive** for SARS-CoV-2: ☐ Yes ☐ No
- Has at least one person living in the home been in **contact with a person** with a **possible/suspected** SARS-CoV-2 infection (COVID-19)? (symptoms listed above) ☐ Yes ☐ No
- Has at least one person living in the home been in **contact with a person** with a **proven** SARS-CoV-2 infection (COVID-19)? ☐ Yes ☐ No

Sports, sleep and food habits during the lockdown :

- Did the young with diabetes feel that he/she was practising sport (if the young is not old enough to give his/her feelings, the feelings of his/her parents are necessary) :
  - ☐ More often ☐ Less often ☐ In the same way ☐ Do not know
- During the lockdown, **how many times a week** did the young with diabetes practise sport?
  - ☐ 0-1 ☐ 2-3 ☐ 4-6 ☐ 7 or more (record each session as lasting at least 30 minutes)
- Has the young with diabetes changed his/her sleep pattern? ☐ Yes ☐ No
  - If **yes**, specify average bedtime and wake-up time during lockdown :
    - Bedtime : _____h_____ Wake-up time : _____h_____
- Did the young with diabetes feel he/she was eating a balanded diet? (if the young is not old enough to give his/her feelings, the feelings of his/her parents are necessary) ☐ Yes ☐ No
- Did the young with diabetes have the impression of increasing his/her carbohydrate intake (bread, pasta, rice, potatoes, semolina, sweet products, soft drinks, juices, etc.) at mealtimes ? (if the young is not old enough to give his/her feelings, the feelings of his/her parents are necessary) ☐ Yes ☐ No
- **How many times a week** did the young with diabetes snack between meals? ☐ 0 ☐ 1-3 ☐ 4-7 ☐ 8 or more

Diabetes survey during the lockdown :

- Did the young with diabetes feel that he/she has **a poorer glycemic control**? (if the young is not old enough to give his/her feelings, the feelings of his/her parents are necessary) ☐ Yes ☐ No
- Did the young with diabetes feel that he/she has an increase of **hypoglycemia**? (if the young is not old enough to give his/her feelings, the feelings of his/her parents are necessary) ☐ Yes ☐ No
- Did the young with diabetes feel that he/she has an increase of **hyperglycemia**? (if the young is not old enough to give his/her feelings, the feelings of his/her parents are necessary) ☐ Yes ☐ No
- Have you taken the initiative to contact (by e-mail or telephone) your general practitioner or specialist? ☐ Yes ☐ No
