# Supplementary material for: Impact of lockdown on children with type-1 diabetes: returning to the community was associated with a decrease in HbA1c
Source: Front Pediatr. 2023 Dec 20;11:1245861. doi: 10.3389/fped.2023.1245861 (PMC10769491; doi:10.3389/fped.2023.1245861)
Supplement: Supplementary file 2 [file Datasheet2.pdf]

Impact of lockdown on children with type-1 diabetes: case report form

**Identification :** **Initials :** Last name: ___ First name: ___ Birth date __ __ / __ __ / __ __ __ __
Gender ☐ M ☐ F Current weight: ____.___ kg
Date of consultation / hospitalization: __ __ / __ __ / __ __ __ __ (cross out what does not apply)

**Medical history :**
Last weight before lockdown: ____.___ kg Date of this last weight: __ __ / __ __ / __ __ __ __
Date of diabetes diagnosis: __ __ / __ __ / __ __ __ __
Insulin therapy: ☐Pump ☐Multiple injection ☐ Other, specify ____________________
Change of insulin therapy within the last year: ☐ Yes ☐ No If yes, specify the date (MM/YY): __ __ / __ __
Carbohydrate counting: ☐ Yes ☐ No
Continuous glucose monitoring: ☐ FSL ☐ Enlite ☐ Dexcom ☐ None ☐ Other, specify __________________________
Comorbidities ☐ Yes ☐ No If yes, specify __________________________________________
Treatment (other than insulin) ☐ Yes ☐ No If yes, specify ____________________________

**Diabetes data BEFORE lockdown (before March 17th 2020):**
Date of last data collection before lockdown: __ __ / __ __ / __ __ __ __
Total insulin per day before lockdown: ______ IU/kg/d
Number of severe hypoglycemia (with use of glucagon) within 3 months before lockdown: ___
Number of hyperglycemia with ketosis within 3 months before lockdown: ___

**→ CGM data BEFORE lockdown:**
Mean glycemia last 30 days: ____ mg/dL and last 90 days: ____ mg/dL
Proportion of time spent below range / in range / above range (70-180 mg/dL) last 30 days: ____ % / ____ % / ____ %
Proportion of time spent below range / in range / above range (70-180 mg/dL) last 90 days: ____ % / ____ % / ____ %
Average number of flash per day last 30 days: ____ and last 90 days: ____
Proportion of data recorded last 30 days: ____ % and last 90 days: ____ %

**Diabetes data SINCE/AFTER the lockdown (since March 17th 2020):**
Total insulin per day since lockdown: ______ IU/kg/d
Number of severe hypoglycemia (with use of glucagon) within 3 months since lockdown: ___
Number of hyperglycemia with ketosis within 3 months since lockdown: ___

**→ CGM data SINCE/AFTER lockdown:**
Change of CGM : ☐ Yes ☐ No If yes, specify ____________________________________________
Mean glycemia last 30 days: ____ mg/dL and last 90 days: ____ mg/dL
Proportion of time spent below range / in range / above range (70-180 mg/dL) last 30 days: ____ % / ____ % / ____ %
Proportion of time spent below range / in range / above range (70-180 mg/dL) last 90 days: ____ % / ____ % / ____ %
Average number of flash per day last 30 days: ____ and last 90 days: ____
Proportion of data recorded last 30 days: ____ % and last 90 days: ____ %

**Biological data :**
Date and value of last 3 HbA1c **BEFORE** lockdown (**before March 17th 2020**):

- __ __ / __ __ / __ __ __ __ and value of HbA1c: ____ %
- __ __ / __ __ / __ __ __ __ and value of HbA1c: ____ %
- __ __ / __ __ / __ __ __ __ and value of HbA1c: ____ %

Date of first HbA1c at the end of lockdown (**since May 11th 2020**) : __ __ / __ __ / __ __ __ __ and value of HbA1c : _____ %
If PCR SARS-CoV-2 performed: Date: __ __ / __ __ / __ __ __ __ Results : ☐ Positive ☐ Negative
If serology SARS-CoV-2 performed: Date: __ __ / __ __ / __ __ __ __

- Results: IgG: ☐Positive☐Negative☐NA; IgA: ☐Positive ☐Negative☐NA; IgM: ☐Positive☐Negative☐NA

**Lockdown:**

Contact of the patient by the physician by e-mail or telephone during the period of lockdown: ☐ Yes ☐ No

- If yes, for what reason ______________________________________________________________________

Hospitalization since March 17th 2020 : ☐ Yes ☐ No

- If yes, for what reason _________________________
- If yes, specify the date: __ __ / __ __ / __ __ __ __

Change of insulin therapy since March 17th 2020 : ☐ Yes ☐ No

- If yes, for what reason:
  ☐ Chronic diabetes imbalance ☐ Acute diabetes imbalance ☐ Other, specify _____________________
